# De-indexed estimated glomerular filtration rates for the dosing of oral antidiabetic drugs in patients with chronic kidney disease

**DOI:** 10.3389/fphar.2024.1375838

**Published:** 2024-07-04

**Authors:** Maxime Pluquet, Marie Metzger, Christian Jacquelinet, Christian Combe, Denis Fouque, Maurice Laville, Luc Frimat, Ziad A. Massy, Sophie Liabeuf, Solène M. Laville

**Affiliations:** ^1^ Division of Clinical Pharmacology, Amiens University Hospital, Amiens, France; ^2^ MP3CV Laboratory, EA7517, University of Picardie Jules Verne, Amiens, France; ^3^ Centre for Research in Epidemiology and Population Health (CESP), Paris-Saclay University, Versailles Saint Quentin University, Institut National de la Santé et de la Recherche Médicale Unité Mixte de Recherche en Santé 1018, Villejuif, France; ^4^ Biomedecine Agency, Saint-Denis, France; ^5^ Service de Néphrologie Transplantation Dialyse Aphérèse, Centre Hospitalier Universitaire de Bordeaux, Bordeaux, France; ^6^ Institut National de la Santé et de la Recherche Médicale, U1026, Université Bordeaux Segalen, Bordeaux, France; ^7^ Nephrology Department, Centre Hospitalier Lyon Sud, Université de Lyon, Pierre-Bénite, France; ^8^ CarMeN Institut National de la Santé et de la Recherche Médicale 1060, et Association pour l'Utilisation du Rein Artificiel, Université de Lyon, Lyon, France; ^9^ Nephrology Department, Centre Hospitalier Régional Universitaire de Nancy, Vandoeuvre-lès-Nancy, France; ^10^ Adaptation, Mesure et Evaluation en Santé - Approches Interdisciplinaires, Lorraine University, Vandoeuvre-lès-Nancy, France; ^11^ Department of Nephrology, Assistance Publique - Hôpitaux de Paris, Ambroise Paré University Hospital, Paris, France

**Keywords:** chronic kidney disease, diabetes mellitus, kidney function estimation, oral antidiabetic drug, prescribing, pharmacoepidemiology

## Abstract

**Introduction:** Adjusting drug dose levels based on equations that standardize the estimated glomerular filtration rate (eGFR) to a body surface area (BSA) of 1.73 m^2^ can pose challenges, especially for patients with extremely high or low body mass index (BMI). The objective of the present study of patients with CKD and diabetes was to assess the impact of deindexing creatinine-based equations on estimates of kidney function and on the frequency of inappropriate prescriptions of oral antidiabetic drugs (OADs).

**Methods:** The prospective CKD-REIN cohort is comprised of patients with eGFR <60 mL/min/1.73 m^2^. The inclusion criteria for this study were the use of OADs and the availability of data on weight, height and serum creatinine. We compared data for three BMI subgroups (group 1 <30 kg/m^2^; group 2 30–34.9 kg/m^2^; group 3 ≥35 kg/m^2^). Inappropriate prescriptions (contraindicated or over-dosed drugs) were assessed with regard to the summary of product characteristics and the patient’s kidney function estimated with the 2009 Chronic Kidney Disease Epidemiology Collaboration (CKD-EPI) equation, the 2021 CKD-EPI equation, the Modification of Diet in Renal Disease (MDRD) equation, the European Kidney Function Consortium (EKFC) equation, their deindexed estimates, and the Cockcroft-Gault (CG) formula. The impact of deindexing the equations was evaluated by assessing 1) the difference between the indexed and deindexed eGFRs, and 2) the difference in the proportion of patients with at least one inappropriate OAD prescription between the indexed and deindexed estimates.

**Results:** At baseline, 694 patients were receiving OADs. The median BMI was 30.7 kg/m^2^, the mean BSA was 1.98 m^2^, and 90% of patients had a BSA >1.73 m^2^. Deindexing the kidney function estimates led to higher eGFRs, especially in BMI group 3. The proportion of patients with at least one inappropriate prescription differed greatly when comparing indexed and deindexed estimates. The magnitude of the difference increased with the BMI: when comparing BMI group 1 with BMI group 3, the difference was respectively −4% and −10% between deindexed 2021 CKD-EPI and indexed CKD-EPI. Metformin and sitagliptin were the most frequent inappropriately prescribed OADs.

**Conclusion:** We highlight significant differences between the BSA-indexed and deindexed versions of equations used to estimate kidney function, emphasizing the importance of using deindexed estimates to adjust drug dose levels - especially in patients with an extreme BMI.

## Introduction

Diabetes mellitus is a major public health challenge in both developed and developing countries, and the prevalence of this disease continues to increase (Ong et al., 2023). One of the most common comorbidities associated with diabetes mellitus is a deterioration in kidney function, which can lead to the onset of chronic kidney disease (CKD) (American Diabetes Association, 2013). Worldwide, diabetes is the main cause of CKD; people with diabetes are almost twice as likely to have CKD as people without diabetes (Kazancioğlu, 2013; Ene-Iordache et al., 2016). The large number of comorbidities means that drug management in patients with diabetes and CKD is particularly complex (Rossing et al., 2022).

A decline in the glomerular filtration rate (GFR) has a significant impact on the pharmacokinetics and pharmacodynamics of many drugs (Hassan et al., 2009; Nolin, 2015). Appropriate dose level adjustment as a function of the level of kidney function is then needed to avoid drug accumulation and toxicity. However, this adjustment complicates drug therapy management in general and in patients with polypharmacy (as is common in the context of CKD and diabetes) in particular (Alwhaibi et al., 2018; Laville et al., 2018; Schmidt et al., 2019; Kimura et al., 2021).

Before a drug’s dose level can be adjusted to match the patient’s level of kidney function, the latter must be assessed. The GFR can be measured directly using validated, “gold standard” methods, such as measurement of plasma clearance of inulin or a tracer like iohexol. However, these methods are costly and not widely available, and so the GFR is typically estimated by applying an equation. For many years, the Cockcroft-Gault (CG) formula was recommended for the adjustment of drug dose levels in CKD and has been applied during the development of many drugs (Cockcroft and Gault, 1976). In clinical practice, the GFR is usually estimated by applying the 2009 Chronic Kidney Disease Epidemiology Collaboration (CKD-EPI) equation or the Modification of Diet in Renal Disease (MDRD) equation, both of which are recommended for the diagnosis and monitoring of CKD (Levey et al., 1999; Levey et al., 2009; Levin and Stevens, 2014). Both equations are indexed to a standard body surface area (BSA) of 1.73 m^2^. For the adjustment of drug dose levels in patients with CKD, learned societies (Kidney Disease – Improving Global Outcomes (KDIGO) and French National Authority for Health (HAS)) now recommend deindexing kidney function estimates (i.e., not adjusting for the BSA) (Levin and Stevens, 2014; Guide du parcours de soins, 2021). The new CKD-EPI equation introduced in 2021 does not include an ethnic factor (Inker et al., 2021). However, the 2021 CKD-EPI equation was developed for the African-American population in the United States and so should not be used in Europe, where it has been shown to be less accurate than the 2009 CKD-EPI equation (Delanaye et al., 2022; Delanaye et al., 2023a; Delanaye et al., 2023b). During the same period, the European Kidney Function Consortium (EKFC) published a new equation, which was also indexed to a BSA of 1.73 m^2^ (Pottel et al., 2021). The objective of the EKFC formula was to overcome age and ethnicity limitations. Relative to the true GFR measured by inulin clearance, the EKFC formula is just as accurate as the 2009 CKD-EPI equation and the MDRD equation (Delanaye et al., 2022). Indexing to the BSA leads to the over- or underestimation of kidney function in patients with extremely low or extremely high body weight values, respectively. Hence, dose level adjustment according to kidney function is complex for obese patients and especially polymedicated individuals with diabetes mellitus (Laville et al., 2018). Only a few studies have reported on differences between various kidney function estimations for patients with a BSA above 1.73 m^2^ and on the impact of these differences on CKD staging (Vlasschaert et al., 2020).

The literature data show that patients with CKD often receive inappropriate drug prescriptions (i.e., outside the scope of the drug’s marketing authorization (Tesfaye et al., 2017; Laville et al., 2018)), and diabetic patients with CKD are no exception (Muller et al., 2016). However, the proportion of inappropriate prescriptions with regard to the CKD stage varied from one equation to another. We hypothesized that the indexed vs. deindexed difference in the proportion of inappropriate prescriptions increases with the body mass index (BMI) and thus the BSA (well above 1.73 m^2^). When adjusting a dose level, the application of an equation indexed to a standard BSA of 1.73 m^2^ could lead to under-dosing or to incorrect contra-indication of a drug and thus a potential loss of opportunity for the patient.

The objectives of the present study in France were thus to evaluate inter-equation differences in kidney function estimations in a population of patients with CKD treated with oral antidiabetic drugs (OADs). We assessed the differences between BSA-indexed and deindexed creatinine-based estimates of kidney function and the impact of these differences on the frequency of inappropriate prescriptions of OADs [based on the patient’s estimated GFR (eGFR) and the terms of the drug’s European summary of product characteristics (SmPC)].

## Methods

### Study design

CKD-REIN is a French, prospective cohort study of adult (over-18) patients with a confirmed diagnosis of stage 3–5 CKD (eGFR <60 mL/min/1.73 m^2^) who are not on dialysis and have not undergone kidney transplantation. Patients were recruited by 40 nationally representative nephrology facilities. A total of 3,033 patients were included during a routine consultation between 2013 and 2016 and were actively followed up for 5 years. The CKD-REIN study protocol has been approved by the institutional review board at the French National Institute of Health and Medical Research (INSERM; reference IRB00003888) and has been registered at ClinicalTrials.gov (NCT03381950). Details of the CKD-REIN study protocol have been published elsewhere (Stengel et al., 2014).

The present study included 694 patients taking at least one OAD at baseline. We had excluded patients without an OAD prescription at baseline (*n* = 1899) and those with missing data for weight, height, serum creatinine, or prescriptions at baseline (*n* = 80) (Supplementary Figure S1).

### Data collection and management

A specific electronic case report was developed for the CKD-REIN study; it enabled clinical research associates (CRAs) to collect sociodemographic, environmental and clinical information on the study participants at baseline and during the follow-up period. At baseline, the CRAs recorded data gathered in a patient interview and extracted from medical records. Measurement of the patient’s height and weight enabled calculation of the BSA [using the Dubois equation (Du Bois and Du Bois, 1916)] and the BMI. Standard blood and urine tests (i.e., those recommended by the French health authorities for the routine management of CKD) were performed for all patients in their usual medical laboratory. Patients were asked to fill out self-questionnaires on their knowledge of their medications, their adherence to medication (Girerd et al., 2001), and the frequency of consultations with family physicians and specialists in the year preceding inclusion in the study. Patients were classified as having diabetes if 1) this disease was reported in their medical records, 2) the patients were taking glucose-lowering drugs, or 3) HbA1c ≥6.5% or fasting blood glucose ≥7.0 mmol/L or random blood glucose ≥11.0 mmol/L.

At baseline, GFR was estimated using the 2009 CKD-EPI, 2021 CKD-EPI, MDRD and EKFC equations, all of which are indexed to a BSA of 1.73 m^2^ (Levey et al., 1999; Levey et al., 2009; Inker et al., 2021; Pottel et al., 2021). We also estimated kidney function with the CG formula (i.e., the estimated creatinine clearance, expressed in mL/min) (Cockcroft and Gault, 1976), and the BSA-deindexed 2009 CKD-EPI, 2021 CKD-EPI, MDRD and EKFC equations (i.e., the eGFR in mL/min) (Supplementary Table S1). The deindexed equation was obtained by multiplying the indexed equation by the patient’s BSA and dividing by 1.73.

A specific form was used to record the drugs prescribed to the patients during the 3 months prior to study inclusion. Prescriptions were brought to the interview by the patient. For each drug prescription, we recorded the trade name, international non-proprietary name, Anatomical Therapeutic Chemical class, presentation identifier code, unit dose, defined daily dose, pharmaceutical formulation, and administration route.

### Definition of inappropriate drug prescriptions with regard to kidney function

To assess the proportion of inappropriate prescriptions with regard to the patient’s kidney function, we referred to the European SmPC for each OAD as the most detailed source of prescribing guidelines. If a European SmPC was not available, we used the French SmPC. Hence, for each OAD, we screened a SmPC and extracted the contraindications or dosing guidelines related to kidney function. In particular, the kidney function thresholds below which the drug was contraindicated or required dosage adjustment were noted (Supplementary Table S2). A prescription was considered to be inappropriate if, according to the drug’s SmPC, it was contraindicated or prescribed at a too high dose for the patient’s level of kidney function.

Since the right dose of insulin depends on the patient’s measured blood glucose levels, diet, and activity (rather than kidney function), we did not investigate insulin use in the present study. Furthermore, other non-oral antidiabetic agents such as glucagon-like peptide-1 analogs (used by a small proportion of study participants) were not included in our analysis.

### Statistical analyses

The study population consisted of patients taking at least one OAD at baseline. Baseline characteristics were described for the study population as a whole and for each of three BMI groups [group 1 < 30 kg/m^2^; group 2: 30–34.9 kg/m^2^ (obesity: class I); group 3 ≥ 35 kg/m^2^ (severe and morbid obesity: classes II and III)] (Weir and Jan, 2023). Continuous variables were reported as the mean [standard deviation (SD)] or the median [interquartile range (IQR)], depending on the distribution. Categorical variables were reported as the frequency (percentage). Depending on the distribution, we used a chi-squared test or Fisher’s exact test to compare values of categorical variables and an analysis of variance (ANOVA) or the Kruskal–Wallis test to compare values of continuous variables.

We assessed the mean difference between deindexed and indexed equations according to the BMI group with a one-way ANOVA. We calculated the number of patients moving from one CKD stage to another when deindexing the equation used to estimate the GFR. For each of the nine equations used to estimate kidney function (i.e., the 2009 CKD-EPI, 2021 CKD-EPI, MDRD and EKFC equations and their versions deindexed from the BSA, together with the CG formula), we assessed the proportion of patients with at least one inappropriate OAD prescription (i.e., contraindicated or over-dosed) with regard to the patient’s kidney function. We also assessed the numbers of contraindicated and over-dosed prescriptions for each OAD. The difference in the proportion of patients with at least one inappropriate prescription between the indexed and deindexed equations was calculated for each BMI group.

All tests were two-tailed. The threshold for statistical significance was set to *p* < 0.05. Given the low proportion of missing data, we did not adjust for the latter. Statistical analyses were performed using R software (version 4.1.3) (R Core Team, 2022).

## Results

### Characteristics of the study population

Of the 3,033 patients in the CKD-REIN cohort, 694 were analyzed here (Supplementary Figure S1). The median [IQR] age was 71 [65–77], 71% of the patients were men, and 62% had a history of cardiovascular disease (Table 1). For the study population as a whole, the median [IQR] BMI was 30.7 [27.7–34.7] kg/m^2^, the mean (SD) BSA was 1.98 (0.2) m^2^, 90% of patients had a BSA >1.73 m^2^, and 45% had a BSA >2 m^2^. Compared with the other groups, patients with a BMI ≥35 kg/m^2^ were younger, were more likely to be women, and had a higher number of prescription drugs. In this group, the mean (SD) BSA was 2.14 (0.2) m^2^, and 76% had a BSA >2 m^2^.

**TABLE 1:** Baseline characteristics of the study population.

	Total (*N* = 694)	BMI class (kg/m^2^)		
		Group 1 BMI <30 (*N* = 314)	Group 2 BMI 30–34.9 (*N* = 219)	Group 3 BMI ≥35 (*N* = 161)
BMI (kg/m^2^)	30.7 [27.7–34.7]	27.4 [25.5–28.8]	32.1 [31.1–33.5]	38.2 [36.3–41.4]
Men	71%	79%	69%	58%
Age (years)	71.0 [65.3–77.0]	72.0 [67.0–78.0]	71.0 [65.0–76.0]	69.0 [63.0–73.0]
Weight (kg)	87.0 [78.0–99.0]	78.0 [71.0–85.0]	91.0 [84.0–98.0]	108 [99.0–117]
Height (cm)	168 (8.9)	169 (8.5)	168 (8.8)	166 (9.6)
Body surface area (m^2^)	1.98 (0.2)	1.88 (0.2)	2.01 (0.2)	2.14 (0.2)
≤1.73	10%	16%	6%	1%
>1.73	90%	84%	94%	99%
Educational level				
Below high school diploma	70%	67%	73%	73%
High school diploma or higher	28%	31%	25%	25%
* Missing data*	2%	2%	2%	2%
Caucasian	98%	97%	99%	99%
CKD stage (according to the 2009 CKD-EPI equation)				
Stage 2–3A	19%	22%	18%	17%
Stage 3B	42%	40%	46%	39%
Stage 4–5	39%	38%	36%	44%
Serum creatinine level (µmol/L)	162 [135–204]	162 [133–204]	160 [135–197]	164 [135–219]
Smoking status				
Non-smoker	35%	32%	36%	42%
Current	9%	10%	7%	10%
Past	55%	57%	56%	48%
*Missing data*	1%	1%	1%	0%
History of acute kidney injury	22%	20%	21%	27%
*Missing data*	8%	8%	8%	8%
History of cardiovascular disease	62%	64%	64%	53%
*Missing data*	1%	1%	1%	1%
Hypertension	97%	95%	97%	99%
*Missing data*	0.1%	0.3%	0%	0%
Dyslipidemia	90%	88%	91%	91%
*Missing data*	0.1%	0.3%	0%	0%
Treatment compliance				
Good	30%	34%	27%	25%
Minimal	61%	59%	63%	65%
Bad	8%	6%	9%	10%
*Missing data*	1%	1%	1%	0%
Number of prescriptions	10 [8–12]	9 [7–12]	10 [8–12]	11 [9–14]
Number of antidiabetic classes	1 [1–2]	1 [1–2]	1 [1–2]	2 [1–2]
Number of oral antidiabetic drugs	1 [1–2]	1 [1–2]	1 [1–2]	1 [1–2]
Insulin use	39%	30%	43%	49%
Consultations with a family physician, per year				
0 times	2%	1%	3%	1%
1 or 2 times	7%	7%	5%	12%
>2 times	75%	74%	78%	75%
*Missing data*	16%	18%	14%	12%
Consultations with a nephrologist, per year				
0 times	2%	1%	2%	4%
1 or 2 times	57%	61%	53%	55%
>2 times	26%	22%	30%	30%
*Missing data*	15%	16%	15%	11%
Consultations with an endocrinologist, per year				
0 times	29%	31%	32%	24%
1 or 2 times	34%	32%	32%	41%
>2 times	14%	11%	14%	19%
*Missing data*	23%	26%	22%	16%

BMI, body mass index; CKD, chronic kidney disease; CKD-EPI, chronic kidney disease epidemiology collaboration.

### Differences between kidney function equations as a function of the BMI

**FIGURE 1:**
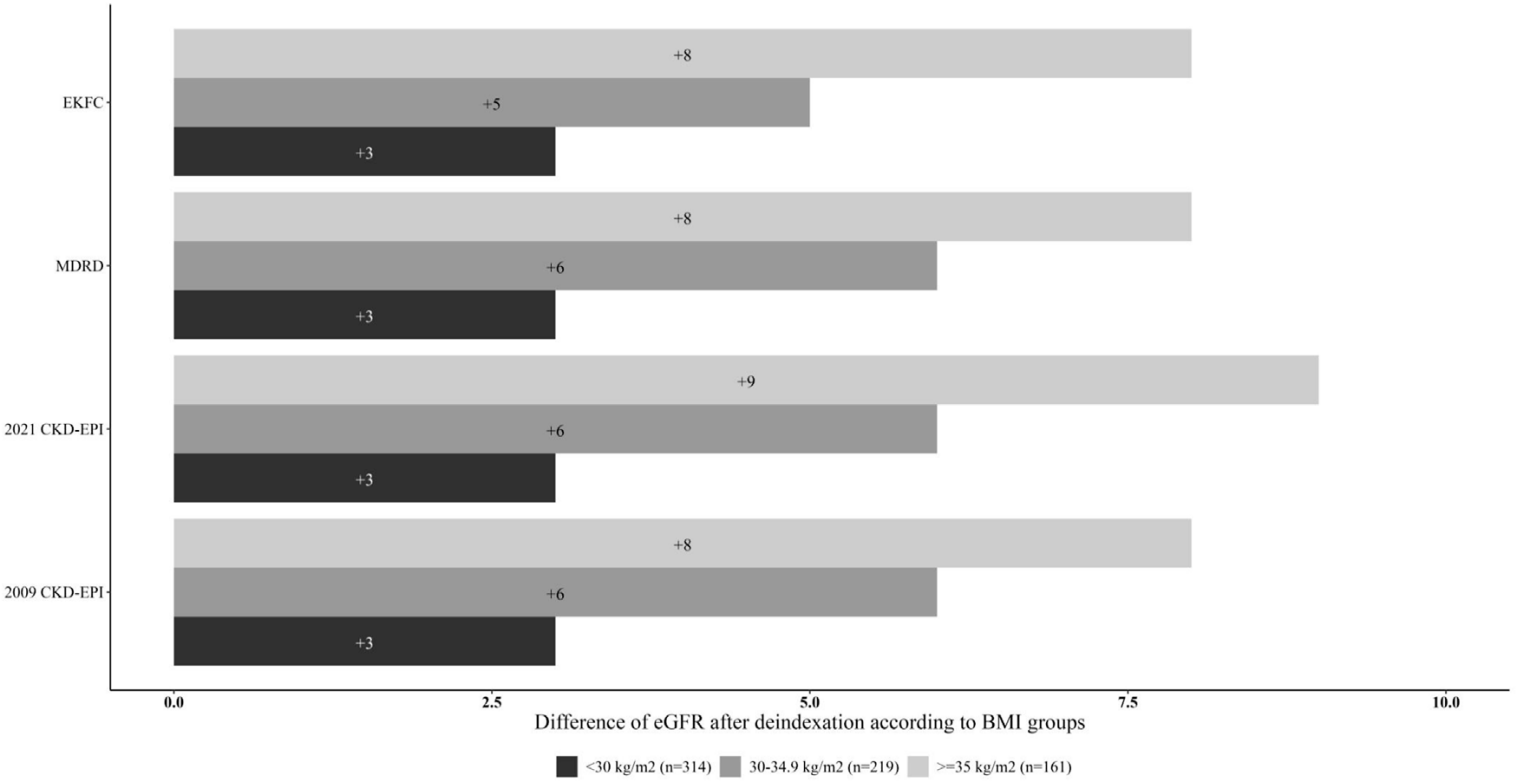
The difference in eGFR between indexed and de-indexed equations, by BMI group.

In general, deindexing an equation led to a higher eGFR in our study population. The difference between deindexed and indexed estimates was significantly higher when the BMI was higher (Table 2; Figure 1). The greatest difference was found with 2021 CKD-EPI, and the smallest difference was found with EKFC.

**TABLE 2:** Differences between deindexed and indexed kidney function estimates according to the equation used, by BMI group.

	Total (*N* = 694)	BMI (kg/m^2^)			*p*-value
		Group 1 BMI <30 (*N* = 314)	Group 2 BMI 30–34.9 (*N* = 219)	Group 3 BMI ≥35 (*N* = 161)	
2009 CKD-EPI	+5.1 (4.7)	+3.2 (3.8)	+5.6 (4.2)	+8.0 (5.4)	<0.001
2021 CKD-EPI	+5.4 (5.0)	+3.4 (4.0)	+5.9 (4.4)	+8.5 (5.7)	<0.001
MDRD	+5.0 (4.6)	+3.2 (3.7)	+5.6 (4.1)	+7.9 (5.2)	<0.001
EKFC	+4.9 (4.6)	+3.0 (3.6)	+5.4 (4.0)	+7.8 (5.2)	<0.001

BMI, body mass index; CKD-EPI, chronic kidney disease epidemiology collaboration; EKFC, European kidney function consortium; MDRD, modification of diet in renal disease.

### The change in CKD stage upon deindexation from the BSA

Upon deindexation of the 2009 CKD-EPI equation, 235 patients moved to a lower CKD stage and 13 moved to a more advanced stage (Table 3). The corresponding values were 241 and 4 for the 2021 CKD-EPI equation, 233 and 9 for the MDRD equation, and 215 and 8 for the EKFC equation.

**TABLE 3:** Number of patients with a change in CKD stage after de-indexation of the equation used to estimate kidney function.

	2009 CKD-EPI	2021 CKD-EPI	MDRD	EKFC
CKD stage 5 → 4	13	11	12	10
CKD stage 4 → 5	1	1	2	1
CKD stage 4 → 3B	84	61	81	82
CKD stage 3B → 4	5	1	3	3
CKD stage 3B → 3A	103	106	108	95
CKD stage 3A → 3B	4	1	2	1
CKD stage 3A → 2	35	63	32	28
CKD stage 2 → 3A	3	1	2	3

CKD, chronic kidney disease; CKD-EPI, chronic kidney disease epidemiology collaboration; EKFC, European kidney function consortium; MDRD, modification of diet in renal disease.

### Inappropriate prescriptions with regard to the kidney function estimates

**FIGURE 2:**
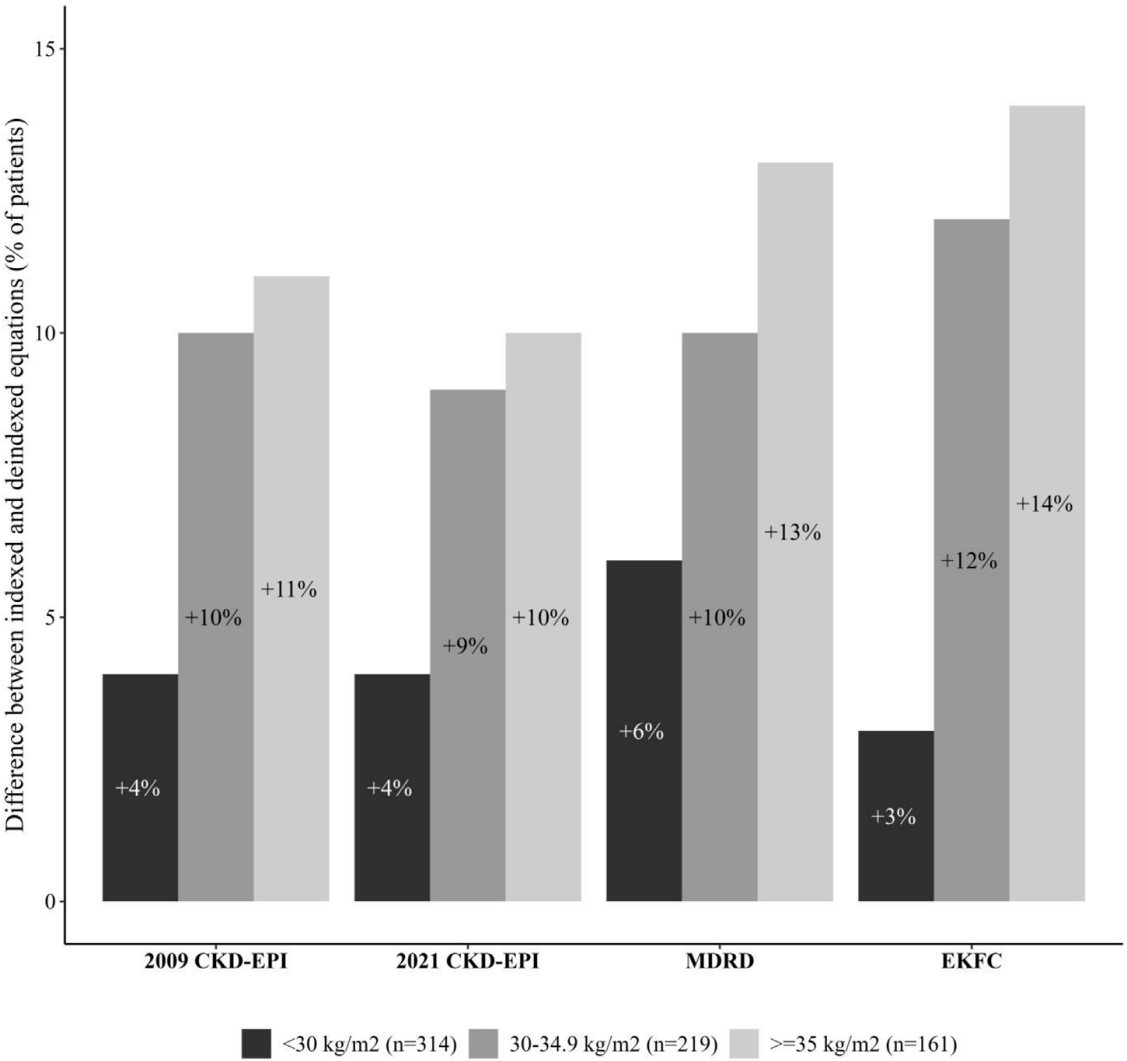
The difference in the proportion of patients with at least one inappropriate OAD prescription between indexed and deindexed eGFR equations, by BMI group.

Overall, the equations which gave the highest proportion of patients with at least one inappropriate OAD prescription were the EKFC (34%), MDRD (32%), and 2009 CKD-EPI (31%) equations. The lowest proportions were found with the CG formula (18%), deindexed 2021 CKD-EPI (20%), deindexed MDRD and deindexed 2009 CKD-EPI equations (23%). In patients in BMI group 1, the highest proportion was found with the EKFC equation (32%), and the lowest was found with the deindexed 2021 CKD-EPI equation (23%) (Supplementary Figure S2A). In patients in BMI group 2, the highest proportion was found with the EKFC equation (36%), and the lowest was found with the CG formula (15%) (Supplementary Figure S2B). In patients in BMI group 3, the highest proportion was found with the EKFC equation (35%), and the lowest was again found with the CG formula (10%) (Supplementary Figure S2C). The indexed vs deindexed difference in the proportion of patients with at least one inappropriate OAD prescription rose with the BMI group: when comparing BMI group 1 with BMI group 3, the difference was respectively 4% and 10% with the 2021 CKD-EPI equation, 4% and 11% with the 2009 CKD-EPI equation, 6% and 13% with the MDRD equation, and 3% and 14% with the EKFC equation (Figure 2). Metformin and sitagliptin were the most frequent inappropriately prescribed OADs (Table 4).

**TABLE 4:** Inappropriate prescriptions according to the summary of product characteristics and the equation used to estimate kidney function.

	ATC class	Number of evaluable prescriptions	Number of inappropriate prescriptions for the indexed equation	Number of inappropriate prescriptions for the deindexed equation
2009 CKD-EPI				
Metformin	A10BA02	231	117	84
Glibenclamide	A10BB01	20	6	2
Glipizide	A10BB07	2	1	1
Gliclazide	A10BB09	98	28	16
Glimepiride	A10BB12	36	5	4
Metformin and sitagliptin	A10BD07	30	17	13
Metformin and vildagliptin	A10BD08	21	15	12
Metformin and saxagliptin	A10BD10	1	0	0
Acarbose	A10BF01	30	4	2
Miglitol	A10BF02	1	1	1
Sitagliptin	A10BH01	68	35	22
Vildagliptin	A10BH02	116	15	14
Saxagliptin	A10BH03	5	3	3
2021 CKD-EPI				
Metformin	A10BA02	231	100	73
Glibenclamide	A10BB01	20	3	2
Glipizide	A10BB07	2	1	1
Gliclazide	A10BB09	98	19	12
Glimepiride	A10BB12	36	5	3
Metformin and sitagliptin	A10BD07	30	16	10
Metformin and vildagliptin	A10BD08	21	14	11
Metformin and saxagliptin	A10BD10	1	0	0
Acarbose	A10BF01	30	3	1
Miglitol	A10BF02	1	1	1
Sitagliptin	A10BH01	68	32	20
Vildagliptin	A10BH02	116	14	14
Saxagliptin	A10BH03	5	3	2
MDRD				
Metformin	A10BA02	231	121	89
Glibenclamide	A10BB01	20	6	2
Glipizide	A10BB07	2	1	1
Gliclazide	A10BB09	98	26	13
Glimepiride	A10BB12	36	5	3
Metformin and sitagliptin	A10BD07	30	18	11
Metformin and vildagliptin	A10BD08	21	15	11
Metformin and saxagliptin	A10BD10	1	0	0
Acarbose	A10BF01	30	4	1
Miglitol	A10BF02	1	1	1
Sitagliptin	A10BH01	68	34	21
Vildagliptin	A10BH02	116	15	14
Saxagliptin	A10BH03	5	3	3
EKFC				
Metformin	A10BA02	231	129	94
Glibenclamide	A10BB01	20	6	2
Glipizide	A10BB07	2	1	1
Gliclazide	A10BB09	98	31	20
Glimepiride	A10BB12	36	6	4
Metformin and sitagliptin	A10BD07	30	19	13
Metformin and vildagliptin	A10BD08	21	15	14
Metformin and saxagliptin	A10BD10	1	0	0
Acarbose	A10BF01	30	4	2
Miglitol	A10BF02	1	1	1
Sitagliptin	A10BH01	68	37	27
Vildagliptin	A10BH02	116	15	14
Saxagliptin	A10BH03	5	4	3

ATC, anatomical therapeutic chemical; CKD-EPI, chronic kidney disease epidemiology collaboration; EKFC, European kidney function consortium; MDRD, modification of diet in renal disease.

## Discussion

Our study of a well-characterized population of patients with CKD and diabetes evidenced significant differences in kidney function estimates when the estimating equations (the 2009 CKD-EPI, 2021 CKD-EPI, MDRD and EKFC equations) were deindexed from the BSA – especially in patients with a high BMI. Deindexation had a noteworthy impact on the proportion of inappropriate OAD prescriptions, based on the patients’ eGFR and the prescribing guidelines in the SmPC; for example, the proportion was 31% with the 2009 CKD-EPI equation and 23% with the de-indexed 2009 CKD-EPI equation. Metformin and sitagliptin were the most frequent inappropriately prescribed OADs.

The clinical management of patients with CKD and diabetes is challenging in many respects. These patients frequently suffer from several comorbidities and are thus often polymedicated (Rossing et al., 2022). Polypharmacy is often defined as the use of five or more drugs per day (Varghese et al., 2023). All of our study participants had stage 3–5 CKD, were diabetic and were taking an OAD, and most had a history of cardiovascular disease, dyslipidemia, and/or hypertension. We found that 98% of the OAD-treated patients with CKD are polymedicated, with a median of 10 prescription drugs taken daily. In a study of nearly 9,000 diabetics, 78% took five or more medications daily (Alwhaibi et al., 2018). In the German Chronic Kidney Disease study, the prevalence of polypharmacy was close to 80% in patients with CKD with an eGFR ≥30 mL/min/1.73 m^2^, 62% in CKD stage 1 patients and 86% in CKD stage 3b patients (Schmidt et al., 2019). In the present study, 55% of the participants were obese. Hence, the BSA in these participants deviated significantly from the standard value of 1.73 m^2^ to which most of the equations used to estimate kidney function are indexed: the mean BSA was 1.98 m^2^ overall and 2.14 m^2^ for the patients in BMI group 3.

A study of 366 obese patients compared the deindexed 2009 CKD-EPI equation, the deindexed MDRD equation, and the CG formula with the measured GFR (determined using plasma clearance of ^51^Cr-EDTA) (Bouquegneau et al., 2016). The deindexed MDRD equation was most accurate (80%), followed by the deindexed 2009 CKD-EPI equation (76%). The CG formula was much less accurate (57%). In a study of more than 30,000 patients suffering from atrial fibrillation, it was found that the creatinine clearance rate calculated with the CG formula in patients with a high BMI was higher than the eGFR from the deindexed 2009 CKD-EPI, which was higher than that estimated with the 2009 CKD-EPI equation (Lyu et al., 2023). We saw the same trend in our study, in which more than half of the participants were obese. The creatinine clearance rate estimated with the CG formula was higher than deindexed eGFRs, which were higher than the BSA-indexed eGFRs. The differences between indexed and deindexed values were greater in the higher BMI groups: between 5 and 6 eGFR units in BMI group 2 and between 8 and 9 units in BMI group 3. We also found that deindexing the eGFR from the BSA moved around a third of patients to a lower CKD stage. A study of 281 obese patients with CKD found that deindexing the 2009 CKD-EPI and MDRD equations reduced the CKD stage for respectively 52% and 51% of the individuals with a BSA above 2.2 m^2^ (Vlasschaert et al., 2020).

Most of the world’s learned societies now recommend deindexing kidney function estimates when adjusting drug dose levels in patients with CKD (Levin and Stevens, 2014). In the context of drug development in general and pharmacokinetic studies and dose assessment in patients with impaired kidney function in particular, the European Medicines Agency (EMA) now recommends expressing eGFR in mL/min, rather than as a value standardized against a BSA of 1.73 m^2^ (Committee for Medicinal Products for Human use (CMPH) - European Medicines Agency, 2015). However, the EMA does not specify which equation to use. The CLEAR study reported that of 2,447 reviewed French SmPCs, 438 recommended a dose level adjustment according to the level of kidney function. Ninety percent of the SmPCs did not specify which estimating equation could or should be used, 9% specified the CG formula, and less than 1% specified the 2009 CKD-EPI equation or the MDRD equation (Berdougo et al., 2020). For the 14 OADs considered in the present study, 13 of the European SmPCs required adjustment of the dose level according to kidney function: three recommended the use of the creatinine clearance rate, six recommended the use of a BSA-deindexed equation (without specifying which one), and four SmPCs did not specify which equation could or should be used.

The literature data show that patients with CKD often receive drug prescriptions that are inappropriate with regard to their kidney function (Tesfaye et al., 2017; Laville et al., 2018; Castelino et al., 2020). In our study, the proportions of patients with at least one inappropriate OAD prescription (with regard to the patient’s estimated kidney function and the prescribing guidelines in the SmPC) were high and ranged from 18% with the CG formula to 34% with the EKFC equation. The deindexed equations tended to give smaller proportions of patients with at least one inappropriate OAD prescription (31% vs 23% upon deindexation of the 2009 CKD-EPI equation; 27% vs 20% upon deindexation of the 2021 CKD-EPI equation; 32% vs. 23% upon deindexation of the MDRD equation; and 34% vs 23% upon deindexation of the EKFC equation). The difference in proportions between indexed and deindexed equations was greater in BMI group 3 than in BMI group 1.

On the same lines, a study of 301 diabetic patients suffering from CKD (the majority of whom were obese; mean ± SD BMI = 31.7 ± 6.8 kg/m^2^), the proportion of inappropriate OAD prescriptions (according to the European Renal Best Practice guidelines (Guideline development group, 2015)) varied considerably from one equation to another: 38% with the CG formula, 46% with the deindexed 2009 CKD-EPI equation, and 54% with 2009 CKD-EPI equation (Muller et al., 2016). Metformin and sitagliptin were the most frequently involved drugs in Muller et al.’s study and in the present study. Hence, the use of BSA-deindexed equations might enable some patients (particularly those who are overweight or obese) to receive drugs that would be contraindicated or would require a dose level reduction if a BSA-indexed equation were used.

In terms of practical consequences, we encourage prescribers and clinical pharmacists to follow the guidelines on adapting dose levels according to kidney function and in particular, to deindex the eGFR from the BSA (Levin and Stevens, 2014). Our present results highlighted significant differences between indexed and deindexed estimates of kidney function and differences in the proportions of patients with inappropriate OAD prescriptions (particularly for patients with a high BMI and therefore a BSA well above 1.73 m^2^). Thus, with the use of a BSA-indexed equation, patients with a very low BMI would potentially be overdosed or receive drug treatments that they should not, and patients with a very high BMI would be denied access to certain drug treatments.

The present study had several strengths. Firstly, we measured differences between BSA-indexed and deindexed estimates of GFR in a population of outpatients with a high BSA, a confirmed diagnosis of CKD and follow-up in nephrology centers. Secondly, our detailed recording of prescribed drugs and dose levels enabled us to investigate the consequence of deindexing on the frequency of inappropriate OAD prescriptions with regard to the patient’s eGFR and the guidelines in the SmPC.

Our study also had some limitations. Firstly, we lack of a gold standard (i.e., the measured GFR) for comparing eGFR values, so we cannot conclusively assert that deindexed equations are better in overweight and obese patients. Secondly, a small proportion of our patients were receiving the most recently approved antidiabetic drugs [such as glucagon-like peptide-1 analogs (*n* = 41)] and no patients were on sodium-glucose cotransporter-2 inhibitors at baseline (as these medications were not marketed yet in France at that time), which were therefore not considered in our analysis. Thirdly, we only considered two variables when estimating the appropriateness of an OAD prescription: the patient’s eGFR and the guidelines in the SmPC. In fact, several other variables can influence the appropriateness of an antidiabetic prescription, such as the degree of glycemia control, the level of glycated hemoglobin, and interactions with other drugs.

Our study of patients with CKD and diabetes in the CKD-REIN cohort enabled us to highlight significant differences between the equations used to estimate kidney function and between the BSA-indexed and -deindexed versions of each equations. We assessed the impact of BSA deindexation on the proportion of patients with inappropriate OAD prescriptions. Our results emphasized the importance of using deindexed estimates of kidney function when adjusting the drug dose level with regard to the patient’s kidney function - especially in individuals with a very low or very high BMI. De-indexation could help prevent certain drugs from being misused or not used at all.

## Data Availability

The data that support the findings of this study are available upon reasonable request by contacting the CKD-REIN study coordination staff at ckdrein@inserm.fr.

## Group members of CKD-REIN study

### Steering committee and coordinators

The CKD-REIN Study Group steering committee and coordinators include: Natalia Alencar de Pinho, Dorothée Cannet, Christian Combe, Denis Fouque, Luc Frimat, Aghilès Hamroun, Yves-Edouard Herpe, Christian Jacquelinet, Oriane Lambert, Céline Lange, Maurice Laville, Sophie Liabeuf, Ziad A. Massy, Marie Metzger, Pascal Morel, Christophe Pascal, Roberto Pecoits-Filho, Joost Schantsra, Bénédicte Stengel.

### Investigators

Alsace: Professors T. Hannedouche and B. Moulin (CHU, Strasbourg), Dr. A. Klein (CH Colmar), Aquitaine: Professor C. Combe (CHU, Bordeaux), Dr. J. P. Bourdenx (Clinique St Augustin, Bordeaux), Dr. A. Keller, Dr. C. Delclaux (CH, Libourne), Dr. B. Vendrely (Clinique St Martin, Pessac), Dr. B. Deroure (Clinique Delay, Bayonne), Dr. A. Lacraz (CH, Bayonne), Basse Normandie: Dr. T. Lobbedez (CHU, Caen), Dr. I. Landru (CH, Lisieux), Ile de France: Professor Z. Massy (CHU, Boulogne – Billancourt), Professor P. Lang (CHU, Créteil), Dr. X. Belenfant (CH, Montreuil), Professor E. Thervet (CHU, Paris), Dr. P. Urena (Clinique du Landy, St Ouen), Dr. M. Delahousse (Hôpital Foch, Suresnes), Languedoc – Roussillon: Dr. C. Vela (CH, Perpignan), Limousin: Professor M. Essig, Dr. D. Clément (CHU, Limoges), Lorraine: Dr. H. Sekhri, Dr. M. Smati (CH, Epinal), Dr. M. Jamali, Dr. B. Hacq (Clinique Louis Pasteur, Essey-les-Nancy), Dr. V. Panescu, Dr. M. Bellou (Polyclinique de Gentilly, Nancy), Professor Luc Frimat (CHU, Vandœuvre-les-Nancy), Midi-Pyrénées: Professor N. Kamar (CHU, Toulouse), Nord-Pas-de-Calais: Professors C. Noël et F. Glowacki (CHU, Lille), Dr. N. Maisonneuve (CH, Valenciennes), Dr. R. Azar (CH, Dunkerque), Dr. M. Hoffmann (Hôpital privé La Louvière, Lille), Pays-de-la Loire: Professor M. Hourmant (CHU, Nantes), Dr. A. Testa (Centre de dialyse, Rezé), Dr. D. Besnier (CH, St Nazaire), Picardie: Professor G. Choukroun (CHU, Amiens), Dr. G. Lambrey (CH, Beauvais) Provence-Alpes - Côte d’Azur: Professor S. Burtey (CHU, Marseille), Dr. G. Lebrun (CH, Aix-en-Provence), Dr. E. Magnant (Polyclinique du Parc Rambot, Aix-en-Provence), Rhône-Alpes: Professor M. Laville, Professor D. Fouque (CHU, Lyon-Sud) and L. Juillard (CHU Edouard Herriot, Lyon), Dr. C. Chazot (Centre de rein artificiel Tassin Charcot, Ste Foy-les-Lyon), Professor P. Zaoui (CHU, Grenoble), Dr. F. Kuentz (Centre de santé rénale, Grenoble).
