# Supplementary material for: De-indexed estimated glomerular filtration rates for the dosing of oral antidiabetic drugs in patients with chronic kidney disease
Source: Front Pharmacol. 2024 Jul 4;15:1375838. doi: 10.3389/fphar.2024.1375838 (PMC11255395; doi:10.3389/fphar.2024.1375838)
Supplement: Supplementary file 1 [file Presentation1.pdf]

SUPPLEMENTAL TABLE OF CONTENTS

Table S1: Equations of estimated glomerular filtration rate

Table S2: Prescribing guidelines for oral antidiabetic agents examined in the present study (according to the guidelines in the European or French summary of product characteristics)

Figure S1: Study flow chart

Figure S2: Proportion of inappropriate prescriptions of OAD according to the equation used to estimate kidney function. A. In patients with a BMI$<$30 kg/m^2 (n=314). B. In patients with a BMI between 30 and 35 kg/m^2 (n=219). C. In patients with a BMI$\geq$35 kg/m^2 (n=161)

SUPPLEMENTARY MATERIAL

Table S1: Equations of estimated glomerular filtration rate

**2009 CKD-EPI**<br>eGFR (mL/min/1.73 m²) = 141 x min(Scr/κ, 1)$^{\alpha}$ x max(Scr/κ, 1)$^{-1.209}$ x 0.993^{age} x 1.018 (if female) x 1.159 (if black)<br>κ = 0.7 if female; κ = 0.9 if male<br>α = -0.329 if female; α = -0.411 if male<br>min = minimum of Scr/κ or 1<br>max = maximum of Scr/κ or 1<br>Scr = serum creatinine (mg/dL)
**Deindexed 2009 CKD-EPI**<br>eGFR (mL/min) = 2009 CKD-EPI x BSA / 1.73<br>**2021 CKD-EPI**<br>eGFR (mL/min/1.73 m²) = 142 x min(Scr/κ, 1)$^{\alpha}$ x max(Scr/κ, 1)$^{-1.200}$ x 0.9938^{age} x 1.012 (si female)<br>κ = 0.7 if female; κ = 0.9 if male<br>α = -0.241 if female; α = -0.302 if male<br>min = minimum of Scr/κ or 1<br>max = maximum of Scr/κ or 1<br>Scr = serum creatinine (mg/dL)
**Deindexed 2021 CKD-EPI**<br>eGFR (mL/min) = 2021 CKD-EPI x BSA / 1.73<br>**MDRD**<br>eGFR (mL/min/1.73 m²) = 186 x (Scr x 0.0113)$^{-1.154}$ x age$^{-0.203}$ x 1.21 (if black) x 0.742 (if female) x 0.95 (if creatinine assay calibrated by ID-MS)<br>Scr = serum creatinine (µmol/L)
**Deindexed MDRD**<br>eGFR (mL/min) = MDRD x BSA / 1.73<br>**EKFC**<br>eGFR (mL/min/1.73 m²) = 107.3 / (Scr/Q)$^{\alpha}$ x (0.990$^{(age-40)}$ (if age > 40))<br>Scr = serum creatinine (mg/dL)<br>Q = 0.83 until 50 years old; Q = 0.83 + 0.005 x (age – 50)<br>α = 0.322 quand Scr/Q < 1; α = 1.132 quand Scr/Q ≥ 1
**Deindexed EKFC**<br>eGFR (mL/min) = EKFC x BSA / 1.73<br>**Cockcroft-Gault**<br>Estimated creatinine clearance (mL/min) = 1.23 (ou 1.04 if female) x weight (kg) x (140 – age) / Scr<br>Scr = serum creatinine (µmol/L)

BSA: body surface area, CKD-EPI: Chronic Kidney Disease – Epidemiology Collaboration, eGFR: estimated glomerular filtration rate, EKFC: European Kidney Function Consortium, ID-MDS: Isotope dilution mass spectrometry, MDRD: Modification of Diet in Renal Disease

Table S2: Prescribing guidelines for oral antidiabetic agents examined in the present study (according to the guidelines in the European or French summary of product characteristics)

Antidiabetic drugs	GFR threshold for contraindication (mL/min)	Maximum dose with regard to the GFR (mL/min)	Equation recommended for dose adaptation
Metformin	<30	≥60: 3000 mg<br>45-59: 2000 mg<br>30-44: 1000 mg	BSA-deindexed GFR (mL/min)
Glibenclamide	<30	NA	Not specified
Glipizide	<30	NA	Not specified
Gliclazide	<30	NA	Not specified
Glimepiride	<30	NA	Not specified
Met + sitagliptin	<30	Met: ≥60: 3000 mg<br>Met: 45-59: 2000 mg<br>Met: 30-44: 1000 mg<br>Sit: ≥45: 100 mg<br>Sit: 30-44: 50 mg<br>Sit: <30: 25 mg	BSA-deindexed GFR (mL/min)
Met + vildagliptin	<30	Met: ≥60: 3000 mg<br>Met: 45-59: 2000 mg<br>Met: 30-44: 1000 mg<br>Vil: ≥60: 100 mg<br>Vil: <60: 50 mg	BSA-deindexed GFR (mL/min)
Met + saxagliptin	<30	Met: ≥60: 3000 mg<br>Met: 45-59: 2000 mg<br>Met: 30-44: 1000 mg<br>Sax: ≥45: 5 mg<br>Sax: 15-44: 2.5 mg	BSA-deindexed GFR (mL/min)
Acarbose	<25	NA	Creatinine clearance (mL/min)
Miglitol	<25	NA	Creatinine clearance (mL/min)
Sitagliptin	NA	≥45: 100 mg<br>30-44: 50 mg<br><30: 25 mg	BSA-deindexed GFR (mL/min)
Vildagliptin	NA	≥50: 100 mg<br><50: 50 mg	Creatinine clearance (mL/min)
Saxagliptin	<15 (NR)	≥45: 5 mg<br>15-44: 2.5 mg	BSA-deindexed GFR (mL/min)
Repaglinide	NA	NA	NA

BSA: body surface area, GFR: glomerular filtration rate, Met: metformin, NA: not applicable, NR: not recommended, Sax: saxagliptin, Sit: sitagliptin, Vil: vildagliptin

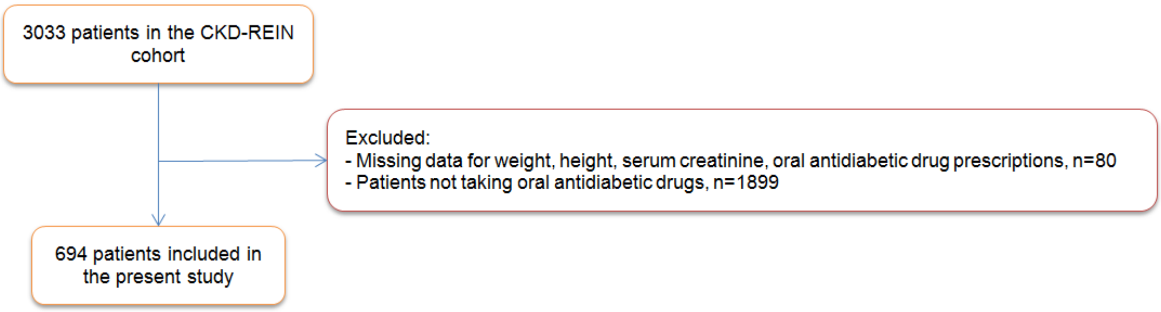

Figure S1: Study flow chart

A. The proportion of patients with at least one inappropriate OAD prescription, for each equation used to estimate kidney function (BMI<30 kg/m², group 1, n=314)

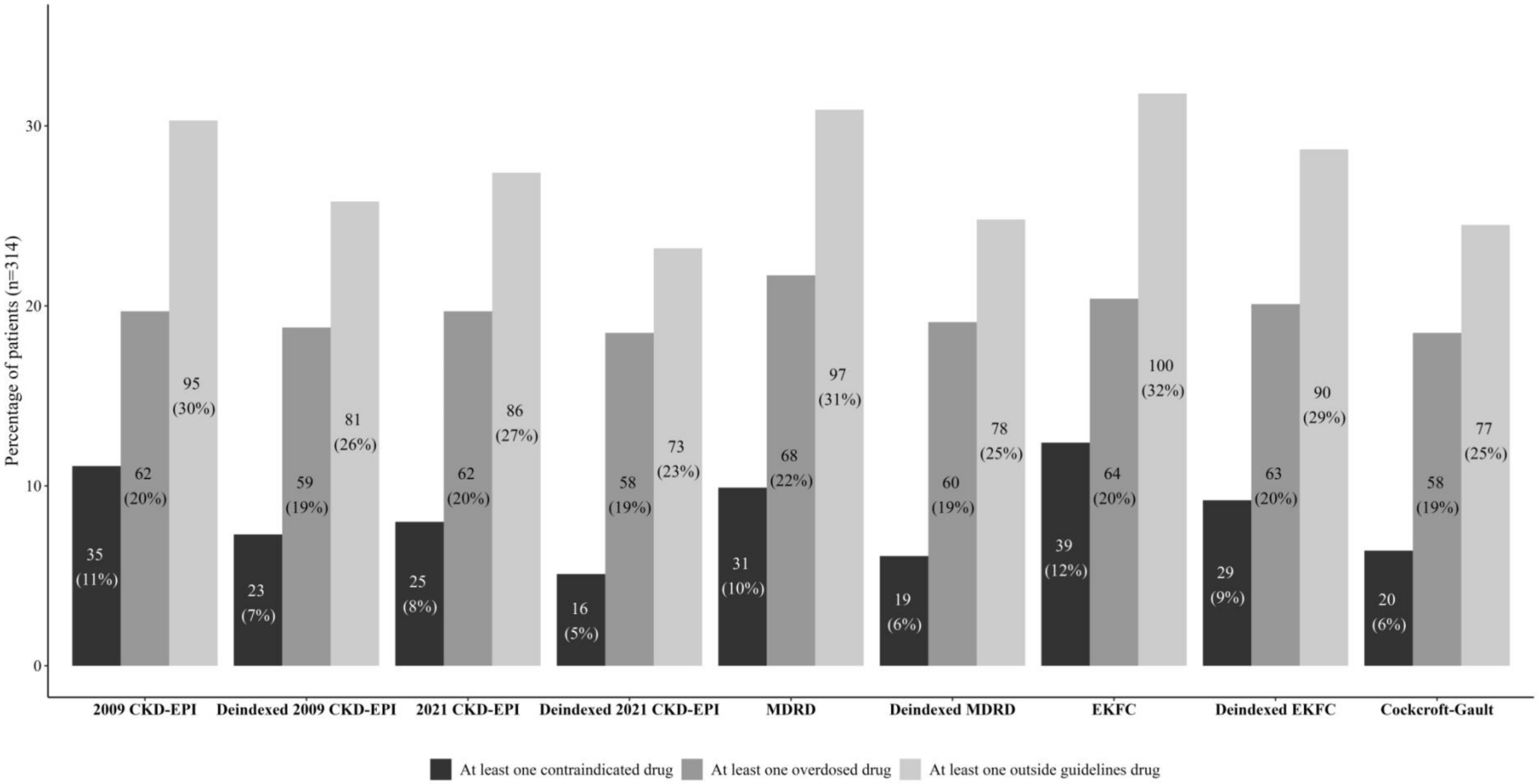

B. The proportion of patients with at least one inappropriate OAD prescription, for each equation used to estimate kidney function (BMI between 30 and 35 kg/m², group 2, n=219)

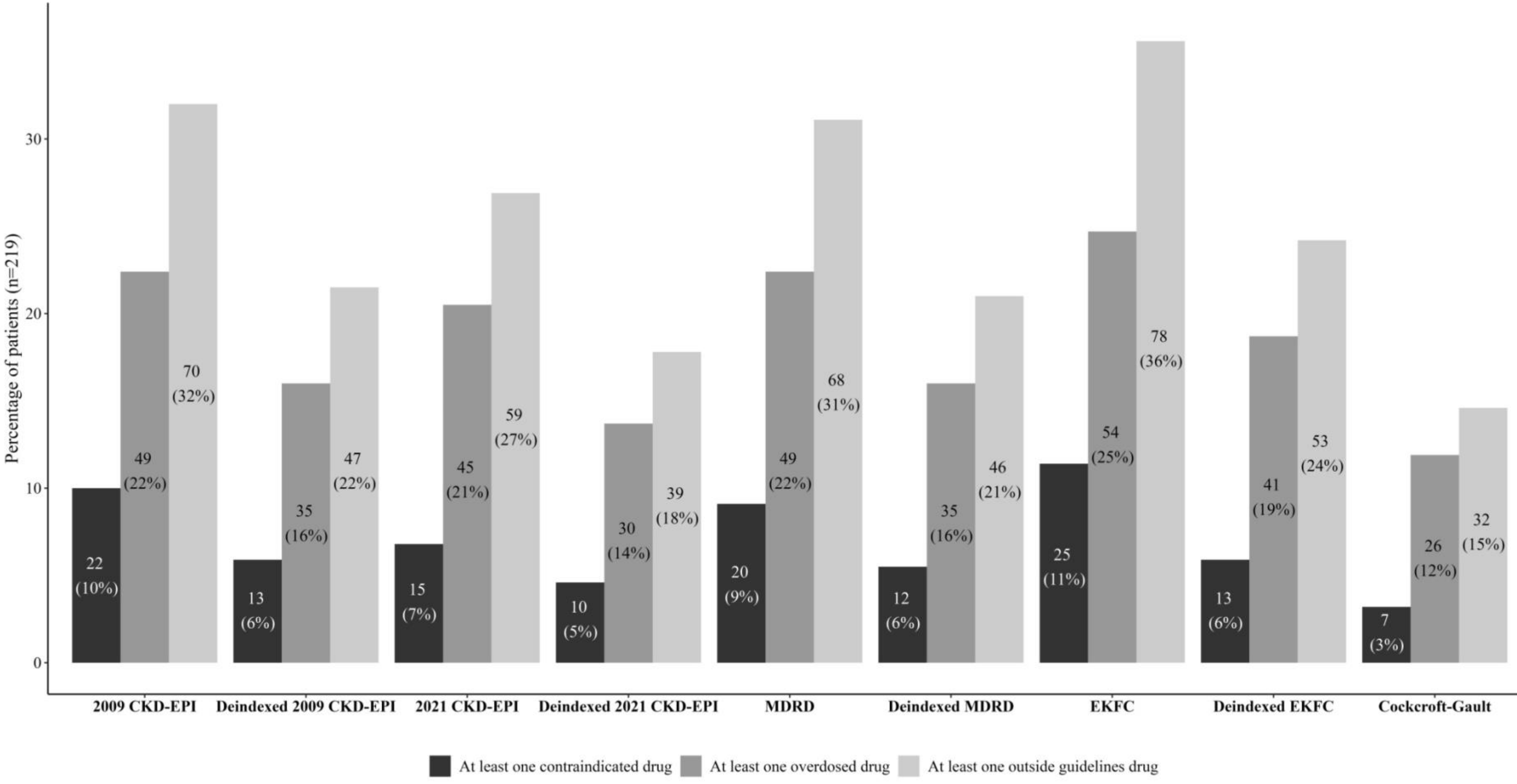

C. **The proportion of patients with at least one inappropriate OAD prescription, for each equation used to estimate kidney function (BMI≥35 kg/m², group 3, n=161)**

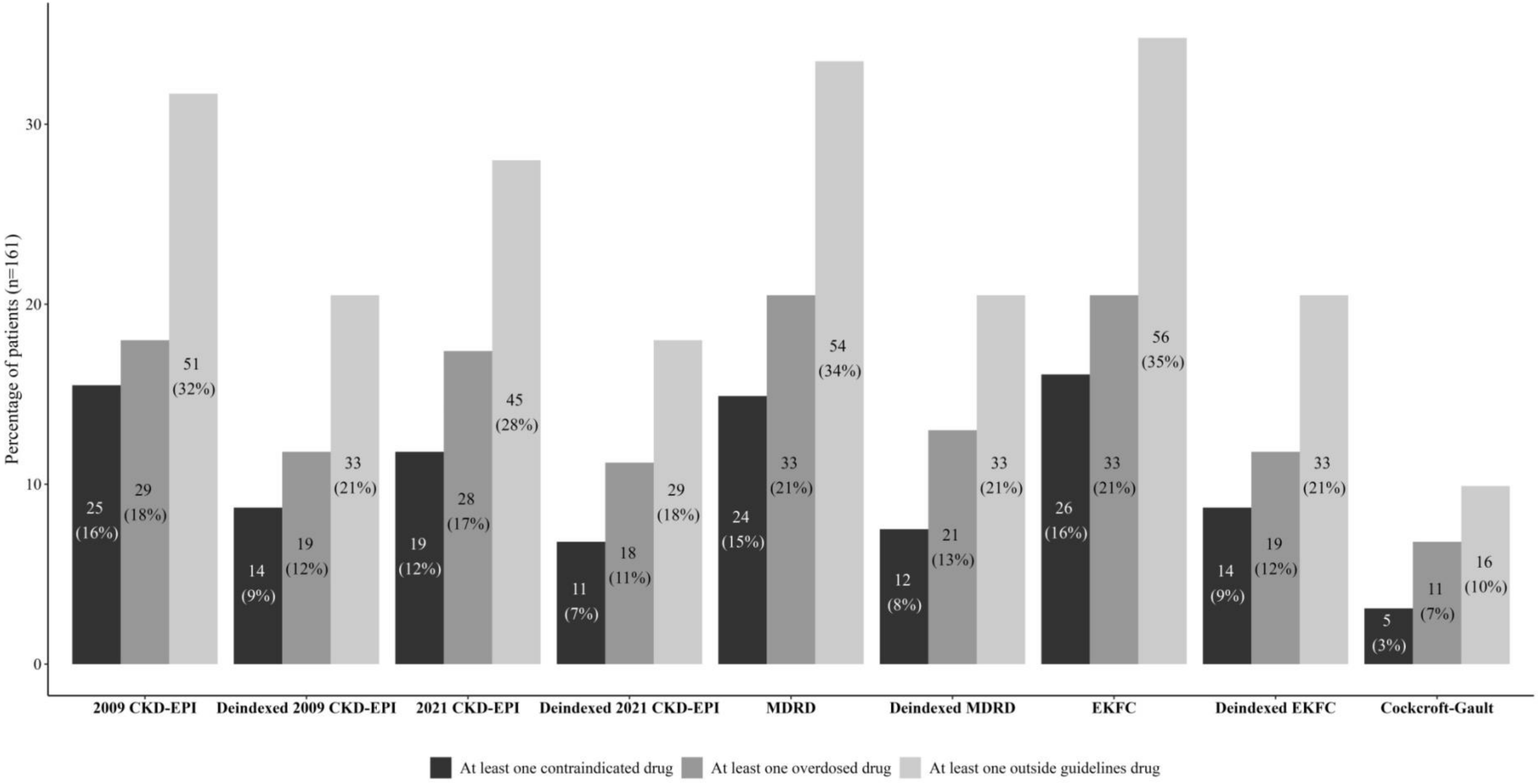

BMI: body mass index, CKD-EPI: Chronic Kidney Disease Epidemiology Collaboration, EKFC: European Kidney Function Consortium, MDRD: Modification of Diet in Renal Disease, OAD: oral antidiabetic drugs

Figure S2: Proportion of inappropriate prescriptions of OAD according to the equation used to estimate kidney function. A. In patients with a BMI<30 kg/m² (n=314). B. In patients with a BMI between 30 and 35 kg/m² (n=219). C. In patients with a BMI≥35 kg/m² (n=161)
